# PReS13-SPK-1247: Activity and damage in juvenile systemic sclerosis- we have to measure them

**DOI:** 10.1186/1546-0096-11-S2-I24

**Published:** 2013-12-05

**Authors:** I Foeldvari

**Affiliations:** 1 Hamburger Zentrum für Kinder - und Jugendrheumatolgie, At Schoen Klinik Eilbek, Hamburg, Germany

## 

Juvenile systemic sclerosis (jSSc) is an orphan disease. There are no established activity and damage indices fort the juvenile form. In the adult form the are prospective studies aiming to validate the activity and damage indices. The assessment of the activity and damage is important in jSSc too to be able to stage the severity of the disease, judge the response to the therapy.

One of the main organ involvement in jSSc is the skin involvement. The modified Rodnan skin score, the pivotal measure of skin involvement. This is not validated prospectively in children with jSSc and it has specific problems in the assessment in a growing child. They are suggestion to adopt it consiedering the Tanner stage of the child. Newer methods like the assessment of the skin with durometer are perhaps more sensitive and have less interrater variation.

The 6 minute walk test, is a pivotal measure in any study to evaluate cardiopulmonary function an required to be used by the licensing agencies to evalute an effect of a new mediaction on pulmonary hypertension. It is not validated either, but recent research established norm values for healthy children as a first to apply it in jSSc patients.

The standardisation of assessment of nailfold capillary changes in jSSc is still evolving, they are few studies looked at the range of changes of the nailfold capillaries in healthy children. They clear changes like capillary loss, which are pathognomic for jSSc.

The composite Medsger index, where all organ involvement are scored and assessed is prospectively in adults with systemic scleroderma is not studied in children. A pilot study recently evaluated a modified form of it.

The new developments will enable us to offer a better care for this seriously sick patients.

## Disclosure of interest

None declared.

